# Editorial: Inflammation and diabetes: unraveling vascular complications and therapeutic innovations

**DOI:** 10.3389/fendo.2026.1999551

**Published:** 2026-10-13

**Authors:** Federico Biscetti, Luis H Eraso, Maria Margherita Rando, Andrea Flex

**Affiliations:** 1 Cardiovascular Internal Medicine Unit, Fondazione Policlinico Universitario A. Gemelli IRCCS, Roma, Italy; 2 Università Cattolica del Sacro Cuore, Roma, Italy; 3 Division of Vascular and Endovascular Surgery, Thomas Jefferson University, Philadelphia, PA, United States

**Keywords:** biomarkers, cardiovascular risk, diabetes, inflammation, MACE (major adverse cardiovascular events)

In 1997, Dr. Attilio Maseri used an elegant metaphor to explain that inflammation was one of the central pillars of cardiovascular medicine that remained undiscovered and undescribed (1). Referring to the often-weak relationship between the extent of coronary stenosis and the myocardial damage observed in acute coronary syndromes, he invited the scientific community to seek new research paradigms rather than continue along familiar, safe paths that were nevertheless insufficient to explain everything. In a way, this is what is needed to describe the dark side of the moon: the hidden and unknown side, without stopping at what we have always been accustomed to seeing.

Inflammation in cardiovascular disease is the dark side of the moon that, in the 1990s, authors such as Peter Libby, Paul M. Ridker, and Attilio Maseri (2), among others, began to explore and reveal. For example, analysis of data from the JUPITER trial clearly showed that the group of patients deriving the greatest benefit from rosuvastatin therapy in terms of cardiovascular events was the one in which, in addition to LDL cholesterol reduction, a reduction in C-reactive protein, one of the most important inflammatory biomarkers, was also achieved (3). Since then, enormous progress has been made, and we have learned that systemic and local inflammation can be a crucial risk factor in the development of major cardiovascular events and beyond. As early as the nineteenth century, Dr. Rudolf Virchow understood that inflammation played a fundamental role in atherosclerosis and vascular disease (4). Over the last 120 years, we have tried to understand the mechanisms underlying these observations.

Diabetes mellitus is intrinsically associated with a chronic pro-inflammatory state that may persist even in patients with apparently adequate glycemic control. Importantly, fasting glucose or glycated hemoglobin levels do not fully capture this inflammatory burden and may contribute to the development and progression of microvascular and macrovascular complications through mechanisms that are, at least in part, distinct from sustained hyperglycemia and glycation-mediated tissue injury. Thus, patients with fasting glucose and glycated hemoglobin values within recommended target ranges may still exhibit persistent inflammatory activation and residual vascular risk. This phenomenon likely reflects the contribution of multiple interacting pathways, some well characterized and others still incompletely understood. Glycemic variability itself may represent one such mechanism, as postprandial glucose excursions can promote the formation of advanced glycation end products and reactive oxygen species, thereby amplifying inflammatory signaling, endothelial dysfunction, and atherosclerotic plaque instability (5). Visceral fat, common in these patients, may also promote the production of pro-inflammatory cytokines such as tumor necrosis factor-alpha and interleukin-6 (6, 7). In addition, intestinal dysbiosis and alterations in the gut microbiota in these patients may induce translocation of inflammatory mediators such as lipopolysaccharide (8). These are only some of the mechanisms through which diabetes may increase inflammatory levels, and none of them can be detected by classic metabolic parameters such as fasting glucose and glycated hemoglobin.

Inflammation in patients with diabetes is certainly one of the factors underlying their increased risk of developing cardiovascular complications (9). For this reason, research and the development of useful risk biomarkers are becoming increasingly important for stratifying patient risk and designing personalized treatments and follow-up strategies (10). In this Research Topic, the authors seek to address these questions. For example, Zhang et al. showed that an interstitial fibrosis and tubular atrophy score of 2/3 is an important marker of worse outcomes in diabetic kidney disease. Furthermore, Xue et al. showed that impaired peripheral thyroid hormone sensitivity is positively associated with coronary heart disease in type 2 diabetes and that serum albumin may be one of the biological mechanisms involved. In addition, Lin et al. explained that immunometabolic dysregulation underpins the pathological progression of diabetic peripheral neuropathy, providing a comprehensive theoretical foundation for understanding its complex pathology and developing targeted therapeutic strategies.

Taken together, these findings will help us continue to uncover and characterize the hidden side of the moon, ultimately guiding us toward more effective and personalized care for our patients.
